# Single-Visit Endodontics Under Dental Operating Microscopy: A Retrospective Observational Study

**DOI:** 10.7759/cureus.111620

**Published:** 2026-06-27

**Authors:** Navdeep Jethi, Rachana Mishra, Minakshi Bhattacharjee, Ankita Pal

**Affiliations:** 1 Conservative Dentistry and Endodontics, Daswani Dental College and Research Centre, Kota, IND

**Keywords:** dental operating microscope, endodontic retreatment, magnification, mandibular incisors, missed canals

## Abstract

Background

Missed lingual canals in mandibular incisors may contribute to persistent post-endodontic pain and treatment failure. Identification and treatment of these untreated canals during retreatment may improve clinical outcomes. This retrospective observational study aimed to evaluate the occurrence of previously untreated lingual canals in symptomatic, previously treated mandibular incisors and to assess symptom resolution following single-visit nonsurgical retreatment.

Materials and methods

Clinical and radiographic records of 129 previously treated single-rooted mandibular incisors were screened. Thirty symptomatic mandibular incisors from 24 patients meeting the inclusion criteria underwent single-visit nonsurgical retreatment. Existing access cavities were refined with a ribbon-shaped lingual extension to facilitate canal exploration. Canal detection was initially performed under unaided vision and subsequently under dental operating microscope (DOM)-assisted magnification when an additional canal could not be identified. Previously untreated lingual canals were treated during the same visit. Pain intensity was recorded preoperatively and at 6 hours, 24 hours, 48 hours, and 7 days postoperatively using the modified 100mm visual analog scale. Canal detection frequencies and postoperative pain outcomes were summarized descriptively.

Results

Among the 129 screened teeth, 99 (76.7%) teeth exhibited a single-canal configuration, while canal configurations involving two canals were identified in 30 (23.3%) teeth. Previously missed lingual canals were identified in all 30 symptomatic mandibular incisors included in the study. Thirteen (43.3%) canals were detected during initial exploration under unaided vision, while 17 (56.7%) canals were identified only after subsequent DOM-assisted evaluation. Pain intensity decreased following retreatment, with complete or near-complete symptom resolution observed by the seventh postoperative day.

Conclusion

Previously untreated lingual canals were frequently identified in symptomatic mandibular incisors requiring retreatment. Single-visit nonsurgical retreatment addressing these untreated canals was associated with a substantial resolution of pre-existing symptoms. The combination of access cavity refinement and sequential unaided and DOM-assisted examination facilitated identification of additional lingual canals during retreatment.

## Introduction

Endodontic treatment failure is primarily associated with persistent intracanal infection resulting from inadequate debridement or failure to address the entire root canal system [1,2]. Among these factors, missed canals represent one of the most preventable causes of post-treatment disease [3]. Untreated canal spaces allow microbes to stay alive, which makes periapical healing harder and often means that the patient needs to be treated again [1,2].

Mandibular anterior teeth have traditionally been considered to possess a simple root canal anatomy [4,5]. However, contemporary anatomical and cone-beam computed tomography (CBCT) studies have demonstrated a considerable prevalence of additional lingual canals and complex canal configurations in these teeth [4,5]. Such anatomical variations are frequently overlooked during primary treatment, particularly when access preparation and canal location are performed under unaided vision [5].

Factors such as obturation remnants, calcifications, dentinal sclerosis, and altered pulpal floor anatomy further complicate canal identification during endodontic retreatment [6]. These challenges reduce tactile and visual cues, increasing the likelihood of missed canals. The dental operating microscope (DOM), by providing enhanced illumination and magnification, facilitates improved visualization of the pulpal floor and enables more conservative and precise access refinement [7].

Single-visit endodontics has gained increasing acceptance due to reduced chair time, lower risk of interappointment contamination, and improved patient compliance [8]. Nevertheless, concerns persist regarding postoperative pain and flare-ups, especially in retreatment cases involving anatomical complexity [9].

Although the use of DOM has been widely advocated in endodontic practice, most supporting evidence originates from in vitro studies, CBCT-based investigations, or clinical studies focused primarily on posterior teeth. Limited clinical data are available regarding its effectiveness during retreatment procedures in mandibular anterior teeth, particularly under routine clinical conditions [10].

Therefore, the present retrospective observational study aimed to evaluate the occurrence of previously untreated lingual canals in symptomatic mandibular incisors undergoing nonsurgical retreatment and to assess the resolution of pre-existing pain following single-visit retreatment.

## Materials and methods

Study design and ethical approval

This retrospective observational study reviewed clinical and radiographic records from 120 patients, including 129 previously treated mandibular incisors that had undergone nonsurgical endodontic retreatment as part of routine care.

The study protocol was reviewed and approved by the Institutional Ethics Committee (IEC No: DDC/ACAD/ETHICAL COMMITTEE/1626) and was conducted in accordance with the Declaration of Helsinki. Written informed consent permitting the anonymized use of clinical data for research and academic purposes had been obtained from all patients at the time of treatment.

All clinical procedures were finalized before the study's inception, ensuring that no outcomes were influenced or altered for research purposes. No additional interventions, deviations from routine clinical practice, or targeted patient recruitment were conducted for the study's purposes. This retrospective observational study was reported in accordance with the Strengthening the Reporting of Observational Studies in Epidemiology (STROBE) guidelines.

Sample selection

Clinical records and digital periapical radiographs of the previous three years from the undergraduate unit of the college were reviewed. A total of 120 patients comprising 129 previously treated mandibular incisors were retrospectively screened for eligibility. Screening was performed by two independent examiners to identify teeth with clinical symptoms and radiographic features suggestive of untreated lingual canals.

Inclusion criteria consisted of previously root canal-treated mandibular incisors requiring nonsurgical retreatment, radiographic suspicion of missed lingual canals, and availability of complete clinical and postoperative pain records. Complete clinical and postoperative pain records were defined as records containing preoperative findings, treatment details, canal detection documentation, and pain assessments recorded at baseline, 6 hours, 24 hours, 48 hours, and 7 days postoperatively.

Exclusion criteria consisted of vertical or horizontal root fractures, periodontal disease, advanced periapical lesions requiring surgical intervention, and incomplete clinical documentation.

Thirty symptomatic mandibular incisors from 24 patients fulfilled the eligibility criteria and were included in the final analysis. Root canal morphology was determined from preoperative and intraoperative clinical records and radiographs documented during treatment and classified according to Vertucci's classification system.

Canal detection protocol

Retreatment procedures were performed by two postgraduate operators as part of their postgraduate clinical training under faculty supervision. Both operators followed the same departmental protocol for access cavity refinement, canal exploration, DOM-assisted evaluation, instrumentation, irrigation, and obturation under faculty supervision.

All retreatment procedures had been performed in a single visit under local anesthesia and rubber dam isolation as part of routine clinical care in the postgraduate unit. Existing coronal restorations were removed, and access cavities were refined in a ribbon-shaped configuration extending lingually to improve visualization of the pulpal floor and facilitate exploration for a possible lingual canal.

Removal of the existing root canal filling material in buccal canals was performed using ProTaper Universal retreatment files (Dentsply Maillefer, Ballaigues, Switzerland) operated at 500 rpm with an endodontic motor (NSK ENDO-MATE, Tochigi, Japan).

Initial canal orifice detection was performed under unaided vision using an endodontic explorer. When an additional canal could not be identified, the pulpal floor was subsequently re-examined using a DOM under magnification and coaxial illumination.

This sequential approach, consisting of access cavity refinement, initial exploration under unaided vision, and subsequent DOM-assisted evaluation when an additional canal could not be identified, was consistently documented in the clinical records. The protocol ensured thorough canal exploration; however, the individual effects of access refinement and magnification on canal detection could not be separated.

Instrumentation protocol

Following canal identification, biomechanical preparation was completed using one of the following instrumentation systems, selected according to operator preference and clinical judgment: group 1, which included the Hand ProTaper Universal system, and group 2, which included the Rotary ProTaper Next system. Working length determination was performed using an electronic apex locator and confirmed radiographically. Cleaning and shaping were completed using standardized irrigation protocols, and obturation was carried out during the same visit. The choice of instrumentation system was not predetermined by the study design and was not intended as a controlled comparative intervention.

Outcome measures

All included teeth were symptomatic prior to retreatment. Preoperative pain was therefore considered a baseline indicator potentially associated with missed lingual canals.

The primary outcome was the detection of previously untreated lingual canals during retreatment, crucial for identifying potential treatment challenges and improving patient outcomes. The secondary outcome was postoperative pain intensity following retreatment, assessed using the modified 100mm visual analog scale (VAS) recorded preoperatively and at 6 hours, 24 hours, 48 hours, and 7 days postoperatively using a standardized pain assessment form (Appendix). Pain assessment methodology was adapted from previously validated pain measurement approaches [11,12]. These data were obtained retrospectively from the clinical records.

Patients were instructed on the use of the VAS form and recorded their pain scores at 6 hours, 24 hours, 48 hours, and 7 days postoperatively as part of routine clinical care. Postoperative pain was patient-reported using a modified 100mm VAS at 6 hours, 24 hours, 48 hours, and 7 days. Pain scores were recorded by patients on standardized forms and obtained retrospectively from clinical records. Analgesics were prescribed when clinically indicated, whereas antibiotics were prescribed only when warranted by clinical findings. Pain assessment was intended to evaluate the resolution of pre-existing pain following identification and treatment of missed lingual canals.

Statistical analysis

Statistical analysis was performed using Microsoft Excel 365 (Microsoft Corporation, Redmond, WA, USA). As the study was observational and retrospective in nature, only descriptive statistical analyses were performed. Instrumentation systems were selected according to operator preference during routine clinical care and were not assigned according to a predefined study protocol. Therefore, inferential statistical comparisons between instrumentation groups were not performed, and outcomes were summarized descriptively. Categorical variables were presented as frequencies, percentages, and 95% confidence intervals where applicable. Pain scores recorded using the Modified 100mm VAS were summarized as mean ± standard deviation. Canal detection frequencies and postoperative pain outcomes were reported descriptively.

## Results

A total of 129 previously treated single-rooted mandibular incisors from 120 patients were screened for eligibility. Morphological evaluation revealed type I canal configuration as the most common observation. The distribution of root canal morphology according to Vertucci’s classification is presented in Table 1.

**Table 1:** Root canal morphology of screened teeth (n = 129)

Vertucci Canal Configuration	n (%)
Type I (1)	99 (76.7)
Type II (2–1)	1 (0.8)
Type III (1–2–1)	24 (18.6)
Type IV (2)	3 (2.3)
Type V (1–2)	0 (0.0)
Type VI (2–1–2)	1 (0.8)
Type VII (1–2–1–2)	1 (0.8)
Total	129 (100)

**Figure 1:**
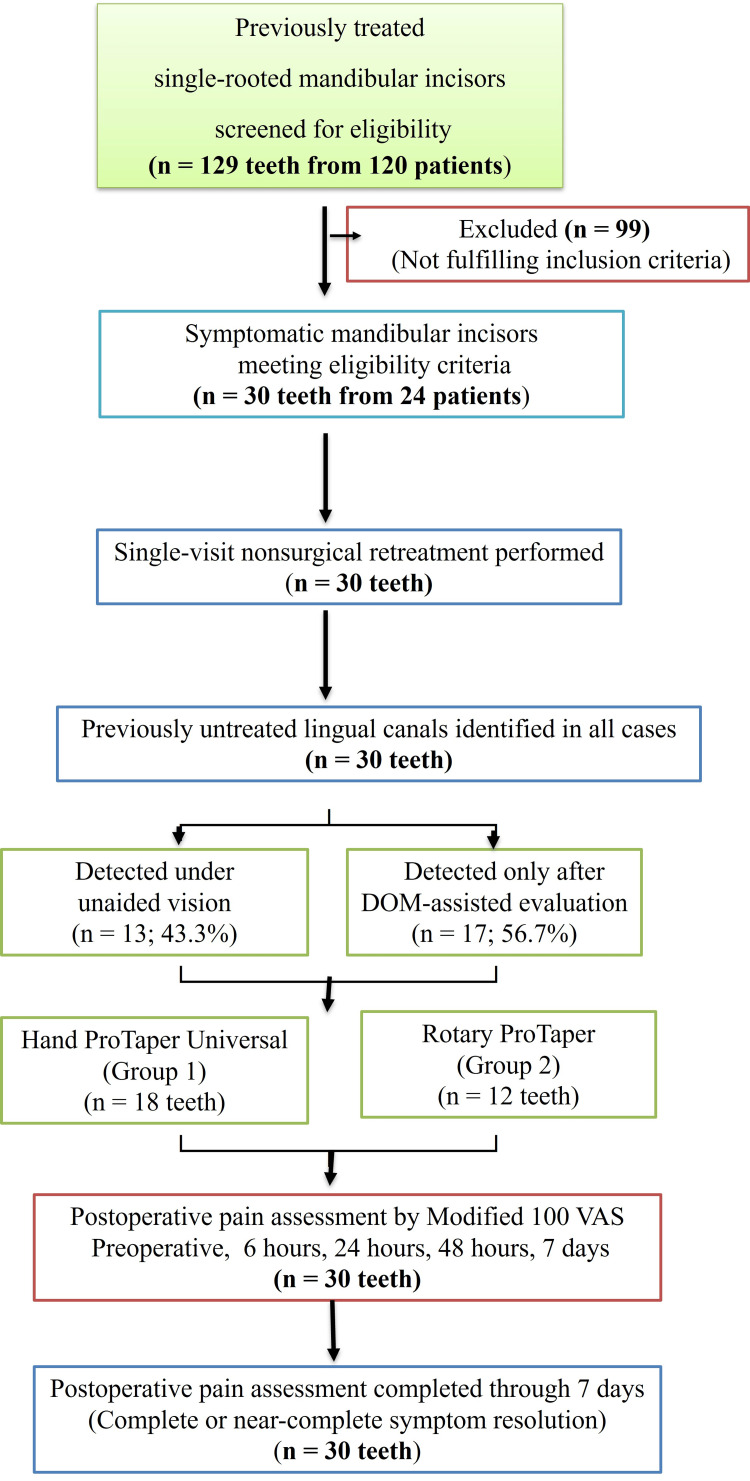
STROBE flow diagram of patient selection, single-visit retreatment, identification of previously untreated lingual canals, and postoperative follow-up. DOM, dental operating microscope; STROBE, Strengthening the Reporting of Observational Studies in Epidemiology; VAS, visual analog scale Figure created using Microsoft PowerPoint 365 (Microsoft Corporation, Redmond, WA, USA).

Among these, 99 (76.7%) teeth exhibited a single-canal configuration, whereas 30 (23.3%) teeth demonstrated a double-canal configuration. Thirty symptomatic mandibular incisors from 24 patients fulfilled the inclusion criteria and underwent single-visit nonsurgical retreatment. The screening, selection, and treatment process is illustrated in Figure 1.

Detection of missed lingual canals

**Figure 2:**
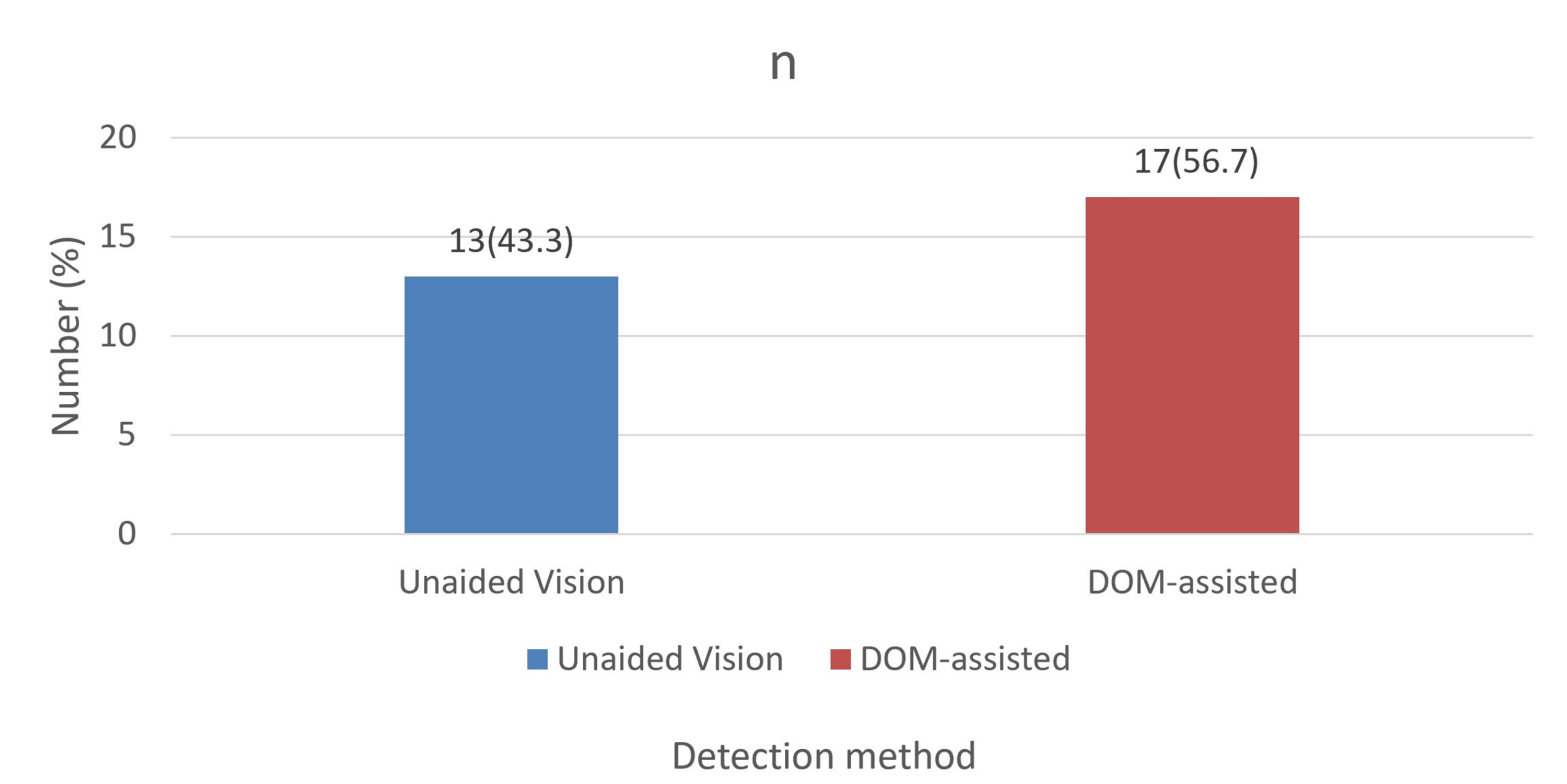
Bar diagram of the detection of untreated lingual canals DOM, dental operating microscope

Sequential canal exploration was performed in all cases, beginning under unaided vision and followed by DOM-assisted evaluation when an additional canal could not be identified. Previously untreated lingual canals were identified in all 30 symptomatic mandibular incisors included in the study. Of these, 13 (43.3%) lingual canals were detected during initial exploration under unaided vision, whereas 17 (56.7%) canals were identified only after subsequent DOM-assisted evaluation (Table 2, Figure 2).

**Table 2:** Detection of previously missed lingual canals during single visit retreatment of symptomatic mandibular incisors (n = 30) Note: sequential canal exploration was performed following refinement of the access cavity into a ribbon-shaped design with lingual extension. Initial canal detection was attempted under unaided vision, followed by DOM-assisted examination when an additional canal could not be identified. DOM, dental operating microscope

Detection Method	n (%)	95% CI
Detected during initial exploration under unaided vision	13 (43.3)	25.6–61.1
Detected only after DOM-assisted evaluation	17 (56.7)	39.0–74.4
Total	30 (100)	—

Postoperative pain outcomes

All included teeth were symptomatic before retreatment. Lower pain scores were observed at successive postoperative evaluations in both instrumentation groups, with complete or near-complete symptom resolution by the seventh postoperative day (Table 3).

**Table 3:** Descriptive postoperative pain scores following single-visit retreatment (modified 100mm VAS) Note: All teeth were symptomatic before retreatment. Pain scores progressively decreased in both groups following retreatment. Pain outcomes are reported according to the instrumentation system documented in the clinical records for descriptive purposes only. SD, standard deviation; VAS, visual analog scale

Time Point	Hand ProTaper Universal (Group 1), Mean ± SD	Rotary ProTaper Next (Group 2), Mean ± SD
Preoperative (baseline)	1.50 ± 1.05	2.04 ± 0.73
6 hours	0.56 ± 0.73	1.00 ± 0.88
24 hours	0.62 ± 0.92	0.54 ± 0.73
48 hours	0.32 ± 0.62	0.18 ± 0.39
7 days	0.00 ± 0.00	0.04 ± 0.20

**Figure 3:**
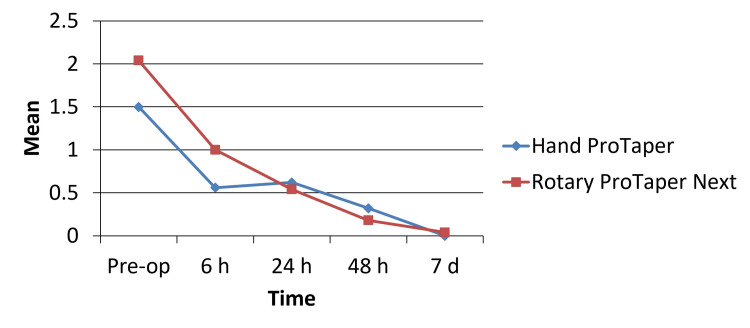
Graphical representation of the resolution of postoperative pain following retreatment

Pain scores decreased progressively across all postoperative evaluation periods in both instrumentation subgroups. Mean pain scores were highest preoperatively and declined substantially at 6, 24, and 48 hours, with complete or near-complete symptom resolution observed by the seventh postoperative day (Table 3, Figure 3). Because instrumentation systems were not allocated according to a predefined study protocol, pain outcomes are presented descriptively without statistical comparison between subgroups.

Regardless of the instrumentation system documented in the original treatment records, pain scores decreased substantially following retreatment, with complete or near-complete symptom resolution by the seventh postoperative day.

## Discussion

The present retrospective observational study evaluated the prevalence of previously missed lingual canals in previously treated symptomatic mandibular incisors and assessed the resolution of pre-existing pain following single-visit nonsurgical retreatment. The use of a DOM facilitated the identification of 56.7% of missed lingual canals that were not initially detected under unaided vision, highlighting the potential value of magnification and enhanced illumination during retreatment procedures [7].

An additional factor that may have contributed to the identification of missed lingual canals was access cavity modification during retreatment [7]. All included teeth had been previously treated under routine undergraduate clinical conditions and subsequently presented with untreated lingual canals. During retreatment, existing access cavities were refined into a ribbon-shaped design with lingual extension, which may itself have improved visualization and access to the lingual canal orifice [7,13,14]. Therefore, the increased detection observed in the present study should likely be attributed to the combined effect of improved access cavity design and DOM-assisted magnification rather than magnification alone.

The prevalence of missed lingual canals in this study aligns with earlier research on complex root canal configurations in mandibular incisors, as reported in previous studies [4,5]. Aldawla and Madfa [4] and Usha et al. [5] demonstrated a substantial prevalence of additional lingual canals and anatomical variations in mandibular anterior teeth. Although these studies focused primarily on anatomical morphology rather than retreatment outcomes, the current findings suggest that such variations are frequently overlooked during primary treatment and may contribute to persistent symptoms requiring retreatment. These observations are corroborated by the CBCT study conducted by Yang et al. [13], which assessed 11,376 mandibular anterior teeth and noted significant variation in root canal configurations based on Vertucci’s classification. Likewise, the systematic review by Dhuldhoya et al. [15] confirmed that mandibular incisors frequently exhibit a second canal, most commonly in a labiolingual orientation, emphasizing the potential for untreated lingual canals during primary endodontic therapy [13,15].

Previous investigations of missed canals have largely relied on CBCT imaging and have predominantly focused on posterior teeth. Mashyakhy et al. [3] and Karobari et al. [16] reported a significant association between untreated canals and persistent apical pathology. Unlike these studies, the present investigation evaluated canal detection under routine clinical conditions using conventional periapical radiographs and microscopic examination. This approach reflects everyday clinical practice and highlights the practical value of DOM-assisted visualization when advanced imaging is unavailable or not routinely indicated [3,16].

The enhanced canal detection achieved with DOM in the present study is consistent with previous evidence supporting the role of magnification in endodontics. Carr and Murgel [7] emphasized that improved illumination and magnification facilitate the identification of subtle anatomical landmarks and additional canals. Similarly, Khalighinejad et al. [17] and Low et al. [18] reported improved outcomes in endodontic treatment performed under microscopic magnification. Although these studies were not restricted to mandibular incisors or retreatment procedures, the present findings extend their observations to symptomatic mandibular anterior teeth and support the routine use of DOM in anatomically challenging cases [17,18].

Postoperative pain assessment was included to evaluate symptom resolution following treatment of previously missed canals. All included teeth were symptomatic before retreatment, indicating unresolved intracanal pathology. Following retreatment, pain scores decreased progressively in both instrumentation groups, with complete or near-complete symptom resolution by the seventh postoperative day. These findings are consistent with those reported by Spohr et al. [9] and Zaneva-Hristova and Borisova-Papancheva [8], who demonstrated favorable postoperative outcomes following effective canal disinfection and retreatment [9,8].

Regardless of the instrumentation system used during retreatment, pain scores decreased substantially following treatment, with complete or near-complete symptom resolution by the seventh postoperative day. As instrumentation systems were selected according to operator preference and were not assigned according to a predefined study protocol, the present study was not designed to compare the effectiveness of different instrumentation techniques. This finding suggests that successful identification and treatment of previously untreated canal anatomy may have a greater influence on clinical outcomes than the instrumentation technique itself [12,19]. Similar observations have been reported by Jethi et al. [12] and Sinha et al. [19], who found that instrumentation methods may influence early postoperative discomfort but have a limited impact on long-term symptom resolution [12,19].

Although CBCT provides superior visualization of complex root canal anatomy, its routine use may be limited by cost, radiation exposure, and accessibility [20]. Antony et al. [20] highlighted the limitations of two-dimensional radiography for detecting anatomical variations, while Honap et al. [21] demonstrated that the combined use of CBCT and a DOM improves identification of complex canal anatomy. Nevertheless, the present findings indicate that DOM-assisted visualization can partially compensate for the limitations of conventional radiography by improving direct clinical identification of untreated canals, thereby enhancing treatment outcomes in routine practice [21].

Limitations of the study include the retrospective design, relatively small sample size, involvement of two operators, absence of routine CBCT confirmation, and lack of long-term radiographic follow-up. Although retreatment procedures were performed according to a standardized departmental protocol, some degree of inter-operator variability cannot be excluded. As treatment procedures reflected routine clinical care, outcomes were presented descriptively without inferential statistical comparisons [22-24].

Despite these limitations, the standardized retreatment protocol, sequential canal detection methodology, and assessment of clinically relevant pain outcomes provide valuable insights into the role of DOM-assisted retreatment in identifying missed lingual canals in mandibular incisors. A major strength of this study was the use of ribbon-shaped access cavity refinement and DOM-assisted evaluation for the identification of previously untreated lingual canals under routine clinical conditions. The study also assessed postoperative pain outcomes following single-visit retreatment.

Future prospective studies incorporating one-year clinical and radiographic follow-up would be valuable in determining whether the short-term symptom resolution observed in the present study translates into sustained periapical healing and long-term treatment success.

Overall, the findings support the use of DOM-assisted retreatment for detecting missed lingual canals in mandibular incisors. Enhanced visualization under magnification facilitates the identification of untreated canal anatomy and may contribute to successful symptom resolution in symptomatic teeth requiring retreatment.

## Conclusions

Within the limitations of this observational study, missed lingual canals were frequently identified in previously treated symptomatic mandibular incisors, suggesting that untreated canal anatomy may be an important contributor to persistent post-treatment symptoms. These findings emphasize the importance of a thorough understanding of root canal anatomy and appropriate access cavity design during primary endodontic treatment, particularly for undergraduate dental training. The combined use of ribbon-shaped access cavity refinement and dental operating microscopy during single-visit retreatment facilitated the identification of previously untreated lingual canals and was associated with marked resolution of preoperative pain in symptomatic mandibular incisors. Further prospective studies with larger sample sizes are warranted to confirm these findings.

## Figures and Tables

**Figure 4:**
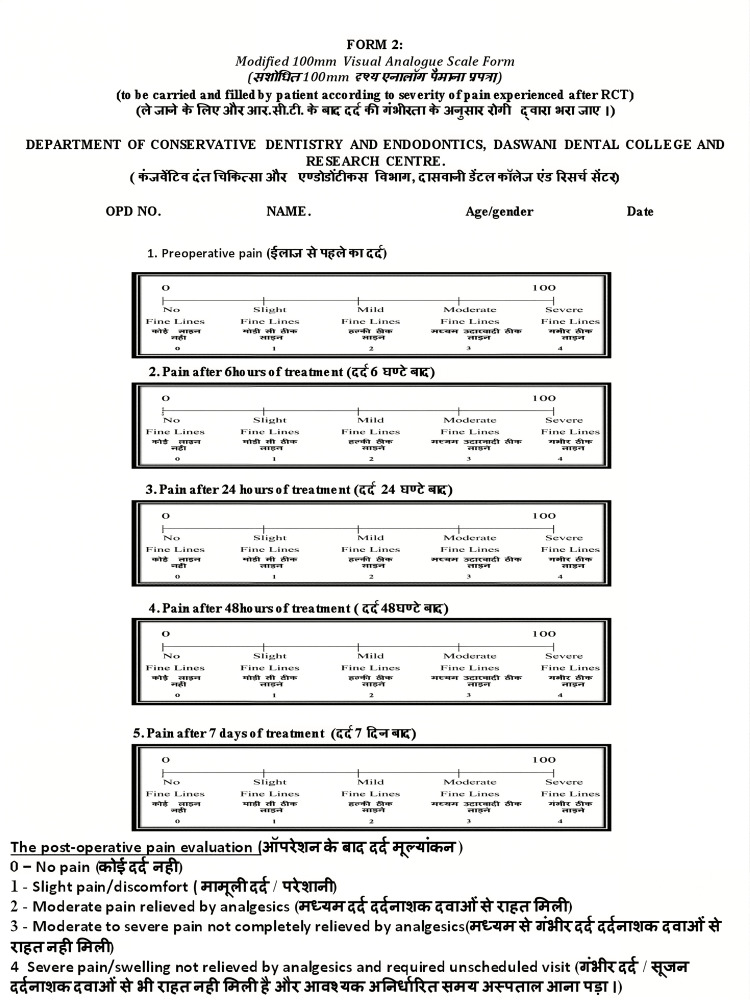
Modified 100mm VAS form used in this study. [12] VAS, visual analog scale
